# Characterizing the utilization of doula support services among birthing people of color in the United States: a scoping review

**DOI:** 10.1186/s12889-024-19093-6

**Published:** 2024-06-13

**Authors:** Emily Kang, Nat’e Stowe, Kelsey Burton, Tiarney D. Ritchwood

**Affiliations:** 1 Duke University, Durham, NC USA; 2 North Carolina Agricultural and Technical University, Greensboro, NC USA; 3 Wake Forest University School of Medicine, Winston-Salem, NC USA

**Keywords:** Doula, Birth coach, Birthing support, Pregnancy, Scoping review, People of color, Post-partum, Disparity, Perinatal

## Abstract

**Background:**

Birthing people of color experience disproportionately higher rates of infant and maternal mortality during pregnancy and birth compared to their white counterparts. The utilization of doula support services may lead to improvements in the birthing experiences of birthing people of color. Yet, the research in this area is sparse. Thus, the purpose of this review is to characterize the research on doula utilization among birthing people of color, identify gaps in the field, and provide recommendations for future research.

**Methods:**

Utilizing PRISMA guidelines, we conducted a scoping review, searching PubMed, PsycINFO, CINAHL, and Google Scholar for peer-reviewed articles published between January 1, 2016, to July 3, 2022.

**Results:**

Twenty-five articles met inclusion criteria. We identified the three themes characterizing included studies: (1) how doulas support (HDS) their clients, (2) doula support outcomes (DSO), and (3) considerations for implementing doula support services (CIDS). Despite doulas being described as agents of empowerment, and providing social support, education, and advocacy, birthing people of color reported low utilization of doula support services and findings regarding their effectiveness in improving birthing outcomes were mixed.

**Conclusions:**

While some studies suggest that doulas may offer important services to birthing people of color, doulas are largely under-utilized, with many birthing people reporting low knowledge of their potential roles during the pre- and post-partum periods. Moreover, few studies were designed to assess intervention effects, limiting our ability to draw firm conclusions. Birthing people of color are at elevated risk for maternal mortality. As such, interventions are needed to support this population and improve outcomes. Our review suggests that, while doulas have the potential to make important contributions to the birthing support team, they are underutilized, and intervention studies are needed to enable estimates of their true effectiveness.

## Background

Birthing people of color in the United States are more likely than their white counterparts to experience adverse birthing-related outcomes, including increased prenatal and postnatal complications, and maternal and infant mortality [1]. For example, compared to their white counterparts, birthing people of color experience worse birth-related events, including hypertension, gestational diabetes, preterm labor, and hemorrhage [2, 3]. The presence of these health conditions before, during, and immediately following pregnancy is associated with poorer health outcomes, including subsequent chronicity [4]. Birthing people identifying as Black, Indigenous, People of Color (BIPOC) are 2–3 times more likely than their white peers to die from pregnancy-related causes [5, 6]. Additionally, non-Hispanic Black/African American birthing people are twice as likely as their non-Hispanic white peers to experience preterm births, increasing health-related risks for both birthing people and their infants [7]. One study found that, compared to 5% of their white counterparts, nearly 10% of Black women and 6% of mixed-race women experienced preterm births prior to 37 weeks’ gestation [8].

The causes of poor birth outcomes among BIPOC birthing people are multifactorial and influenced by multiple levels (e.g., individual, social, community, and societal) and domains, including social determinants of health [9]. Factors associated with poor birth outcomes include structural racism, discrimination, and socioeconomic status [10, 11]. For example, previous research has linked racial disparities in birth outcomes to socio-structural determinants, including the quality of care and discriminatory experiences during the prenatal and labor periods of pregnancy [11, 12]. Additional research has linked discrimination based upon race and socioeconomic status to mistreatment by both support staff and medical providers [13, 14]. Other factors associated with poor pregnancy or birthing outcomes among BIPOC birthing people include structural racism [15–17], being unmarried [18], geographic location [19], comorbid health conditions [4, 10], and lower maternal education [18]. Just as the causes of birthing inequities are multifactorial and span multiple levels, approaches to eliminating known disparities require consideration on multiple levels, spanning policy, practice, advocacy, and engagement [10].

One promising approach to reducing racial/ethnic disparities in pregnancy and birthing outcomes includes leveraging the support of doulas or perinatal support professionals—non-medical or paraprofessionals who guide and support birthing people during pregnancy, labor, birth, and postnatally. Perinatal support is intended to assist birthing people as they transition to parenthood and caregiving and seeks to reduce uncertainty, isolation, and stress [20]. While the role that doulas play in a birthing person’s journey may vary, typical roles have included providing emotional and physical support [21], advocating on behalf of their clients [20], and providing education on topics related to pregnancy, labor, birth, and postnatal care for the birthing person and their infant [22]. While empirical research on this topic remains sparse, some studies have suggested that the expansion of the birthing team to include doulas may help to mitigate negative outcomes attributed to racial inequities in maternal outcomes, such as preterm labor, infections, hemorrhage, gestational diabetes, and hypertension [23, 24]. However, more research is needed to explore the perception, role, and impact of doula utilization on racial disparities in pregnancy and birthing outcomes among BIPOC birthing people.

## Methods

### Aims

This scoping review seeks to characterize the current literature on doula utilization among birthing people of color, including Black/African Americans, identify gaps in the field, and offer recommendations for future directions.

### Design

We conducted a scoping review to explore research on doula utilization among birthing people of color, which was guided by the PRISMA extension for scoping reviews [25]. Since knowledge regarding the extent and depth of evidence on this topic is limited, a scoping review was selected to enable the identification of gaps in the literature and opportunities for further exploration. We followed a 5-step approach to conducting a scoping review, consisting of (1) identifying a research question; (2) balancing the feasibility related to time, funding, and access; (3) selecting articles; (4) extracting data; and (5) reporting results [26].

### Search methods

We searched for peer reviewed articles published between January 1, 2016, to July 3, 2022. We selected these parameters to highlight more recent research on this topic, the increasing interest in the topic based upon the number of published research studies starting in 2016, and time limitations. To identify the articles, we used four databases: PubMed, PsycINFO, CINAHL, and Google Scholar. Assisted by a librarian who developed the search string, we combined key terms using a series of free text and MeSH terms that included ‘doula’, ‘racial inequity’, ‘birth support’, ‘women of color’, ‘minorities’, and ‘birth companion’. Additionally, we used Boolean operators to adjust for spelling variations. We also searched the reference lists of included studies for additional articles. If the title suggested potential study eligibility, we followed our critical appraisal method to determine whether the articles should be included. A full description of the search terms is included in Table 1 (Keywords/Phrases for Searches).

**Table 1:** Search terms and keywords/phrases

“doula”AND “birthing people of color”
“doula” AND “African American women”
“African American women” AND “doula support”
“doula” AND “women of color”
doula minorities
birth support AND women of color
doula
“Birth support” OR “doula” OR “birth coach” AND “women of color” OR “Black women”
doula effect
doula OR birth coach OR birth companion
“community-based doulas” OR “perinatal support professional” OR “monitrice”
birth-companion

### Inclusion/exclusion criteria (setting)

Studies were included in this review if they: (1) had primary outcomes that were relevant to doulas; (2) targeted birthing people of color; and (3) were conducted in the United States. Studies were excluded from this review if they were: (1) not peer-reviewed; (2) published before January 2016; (3) commentaries, reviews, protocols, and reports; or (4) not able to be located or accessed. Moreover, articles were excluded if fewer than 5% of their sample identified as people of color and if the focus was on incarcerated birthing people, as this review seeks to elucidate challenges and opportunities for community-dwelling birthing people of color with autonomy and greater potential to access to doula services. Additionally, for quantitative studies, only one article from a study or dataset was eligible for inclusion, with a preference for those reporting the results of the primary or parent study.

### Search outcomes

After removing duplicates and irrelevant studies following independent title and abstract reviews, we reviewed the full text of the remaining studies. Two members of the study team (EK and NS) reviewed each article, independently, utilizing a screening and selection tool developed for this review that was based upon the inclusion criteria. We resolved disagreements through discussion with the larger team until consensus was met.

### Quality appraisal

We did not formally assess study quality; however, the merits of each study were discussed in detail amongst the team. No studies were excluded due to concerns regarding quality.

### Data extraction

All articles were imported into a reference management software to facilitate file sharing and data organization. For each included study, we extracted information such as author names, publication year, journal name, study title, study design, participant demographic information, and study focus and setting. Studies underwent a comprehensive, full-text review to identify themes, trends, and gaps in the literature.

### Analysis

To describe study findings, we conducted thematic analyses utilizing both inductive and deductive approaches. Utilizing an inductive approach, we examined the results of previous studies, allowing themes to emerge naturally. Concurrently, we utilized a deductive approach to enable a more nuanced analysis of included studies influenced by existing frameworks. By combining these analytic approaches, we could identify commonalities and disparities in the literature more effectively. This process not only allowed us to characterize existing knowledge but also highlighted gaps in the research landscape. Armed with this insight, we were better equipped to propose potential solutions aimed at addressing racial inequities in maternal and infant mortality.

## Results

**Fig. 1:**
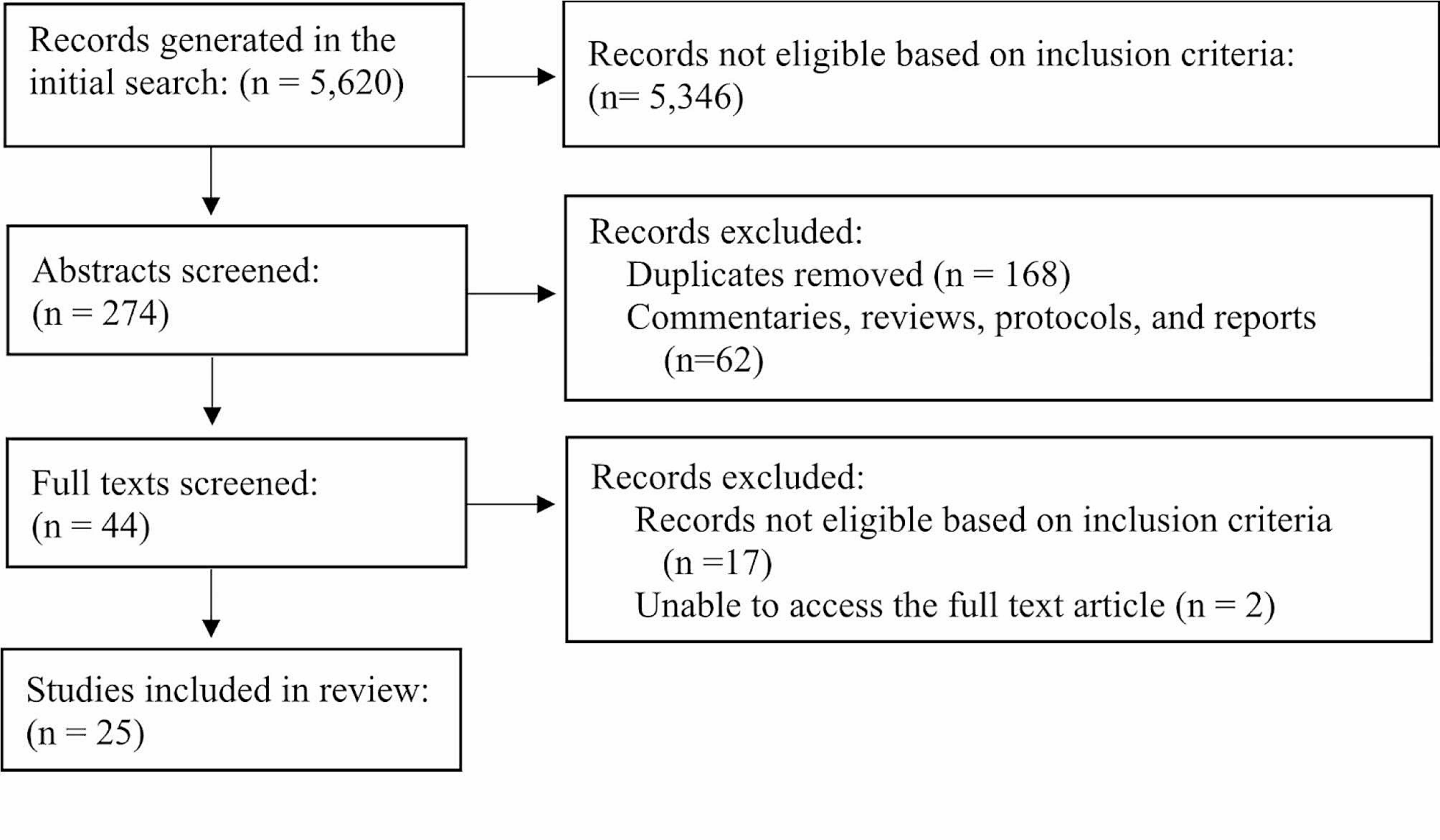
Flow chart of literature search. This figure captures the number of articles identified and screened for the scoping review, as well as those that were excluded, along with the reasons for exclusion

Figure 1 (Flow Chart of Literature Search) presents the PRISMA diagram for this scoping review and reports the total number of studies screened and included at various stages of the screening process. Our initial search identified 5620 articles. After screening the abstract and titles, 5346 articles were excluded, yielding 274 articles. Next, we removed duplicates and non-peer-reviewed or empirical manuscripts and reports, which resulted in 44 studies being selected for full-text screening. Of these, only 25 articles were included in this review.

Tables 2, 3 and 4 present the characteristics of studies included in this review. Of the 25 articles, most were qualitative (*n* = 13), consisting of individual interviews or focus groups discussions. This was followed by retrospective comparison analysis (*n* = 5), cross-sectional survey data analysis (*n* = 2), pre- and post-intervention comparison analysis (*n* = 2), randomized control trial (*n* = 1), quasi-experimental study (*n* = 1), and longitudinal analysis (*n* = 1). Regarding racial and/or ethnic identity among birthing people of color, Black/African Americans, Latinas/Hispanics, and Asians/Pacific Islanders were most studied. Other demographic information reported in some studies included socioeconomic variables, such as health insurance status, household income, marital status, nationality, educational status, and primary spoken language. Geographic diversity was lacking, with all studies focused on populations residing in urban settings.

**Table 2:** How doulas support birthing people of color

Author (year)	*n*	Study Population	Study Design	Doula Support Roles
Attanasio et al., 2021	27	− Women who were pregnant and parenting a child < age 2 (41% white, 30% Black/African American, and 41% Hispanic/Latino) − Women who had received doula training (*n* = 4) − Individuals who had provided support during a birth in the previous 2 years (*n* = 5)	Qualitative Focus Groups	Advocacy Education
Collins et al., 2023	25	− Black/ African American women at least 18 years of age	Qualitative Interviews	Relationship Building Agency & Empowerment
Hansen et al., 2021	11	− Black/African American women between the ages of 25–36 years old who have given birth at least once in North Florida	Qualitative Cross-sectional	Advocacy Education Relationship Building
Guerra-Reyes & Hamiliton, 2017	28	– Self-reported African American nurse-midwives, lay midwives and birth assistants	Qualitative	Advocacy Education Agency & Empowerment
Kathawa et al., 2022	8	− Black/African American and Biracial/ Multiracial doulas between 21–47 years of age	Qualitative Interviews	Relationship Building Agency & Empowerment
Kozhimannil et al., 2016	13	− Low-income pregnant women (38.5% Black/African American, 30.7% African, 15.4% Native American, and 15.4% white women)	Qualitative group discussion	Education Agency & Empowerment
LaMancuso et al., 2016	28	− 14 Karen Refugee women between the ages of 18–41 years old − 8 Karen doulas, community leaders, and interpreters, and 6 clinic representatives	Qualitative interviews	Advocacy
Coley & Nichols, 2016	26	− 20 pregnant adolescents/adolescent mothers between the ages of 15 to 19 years old (65% Black/African American, 5% Biracial, 5% Hispanic, 5% Asian, and 20% white) − Four Black/African American doulas and two white doulas	Qualitative Interviews	Relationship Building
Hans et al., 2022	121	− Adolescent Black/African American girls (13–21 years of age)	Qualitative Interviews	Education
Kett et al., 2022	18	− Black/African American, Hispanic/Latina/Latinx, Indigenous/Alaska Native, and LGBTQIA + doulas providing services to non-white underserved communities	Qualitative Focus Groups and Interviews	Advocacy Education Relationship Building Agency & Empowerment
Wint et al., 2019	10	− 5 Black/African American, 1 Hispanic, 1 Asian or Pacific Islander, 1 Biracial, and 2 white. Six participants had five or more years of doula practice	Qualitative Interviews	Relationship Building
Van Eijik et al., 2022	16	− Executive directors, program directors, or other high-level individuals − Training and certifying organizations, hospitals, and community-based doula organizations in underserved communities	Descriptive (Qualitative)	Advocacy

*Note*: BPOC- Birthing people of colour

**Table 3:** Impact of doulas support on birthing outcomes among birthing people of color

Author (year)	n	Study Population	Study Design	Preterm	Epidural	Caesarean	Breastfeeding	Infant education	Respectful care
Furman et al., 2016	602	Underserved BPOC (85.4% Black/African American)	Quantitative	NA	NA	NA	Significant	NA	NA
Hans et al., 2018	312	Young pregnant BPOC (45% Black/African American and 38% Latina/Hispanic)	Quantitative	Not significant	Significant	Not significant	Significant	Significant	NA
Louis-Jacques et al., 2021	121	Low-income BPOC (63.6% Hispanic, 26.4% Black/African American, and 22.3% white, 15.7% other)	Quantitative	NA	NA	NA	Significant	NA	NA
Kozhimannil et al., 2016	67082	Nationwide Inpatient Sample (22.9% Black, 48.1% White, 10.7% Hispanic, and 2% Asian)	Quantitative	Significant	NA	Significant	NA	NA	NA
Mallick et al., 2022	1977	Participants receiving Medicaid (50.2% Hispanic/Latina, 15.4% Asian/Pacific Islander, 4.3% Black/African American, and 27% white)	Quantitative	NA	NA	NA	NA	NA	Significant
Mosely et al., 2021	9136	Refugee BPOC (62.2% Black)	Quantitative	Not Significant	NA	Not significant	Significant	NA	NA
Thomas et al., 2017	489	Participants mostly receiving Medicaid (84% Black)	Quantitative	Significant	NA	Not significant	NA	NA	NA
Thurston et al., 2019	120	Participants mostly receiving Medicaid (60 Black/African American, 55 white women)	Quantitative	Not significant	Significant	Significant	Significant	NA	NA
Van Zandt et al., 2016	1511	144 Refugees and 215 Non-English speakers (27.5% Black/African American, 13.9% Asian, 32.6% white, 11.7% Hispanic, and 6.9% other racial/ethnic group)	Quantitative	Not significant	Significant	Not significant	Significant	NA	NA

*Note*: BPOC- Birthing people of colour

**Table 4:** Recommendations for implementing a doula support program for birthing people of color

Author (year)	Journal	*n*	Study Design
Marshall et al., 2022	Maternal and Child Health Journal	46	Qualitative
Morton et al., 2018	Birth: Issues in Perinatal Care	2781	Quantitative
Sperlich et al., 2019	Social Work in Health Care	627	Quantitative
Wen et al., 2016	Children and Youth Services Review	246	Quantitative

We grouped studies into three categories based on shared themes and/or reported outcomes: (1) How doulas support (HDS, *n* = 12), (2) doula support outcomes (DSO, *n* = 9), and (3) considerations for implementing doula support services (CIDS, *n* = 4). Articles focused on HDS explored perceptions of the ways in which doulas assist birthing people of color. Moreover, some articles in this category shared suggestions for approaches to leveraging doula support services to improve maternal and/or infant outcomes. Articles focused on DSO explored pregnancy-related outcomes among birthing people based upon whether they received doula support services. Lastly, articles focused on CIDS, though more variable, were linked by an emphasis on adjustments and changes needed to effectively implement a doula support program. While some articles fit in multiple categories, each article was assigned to the category to which it was best aligned based upon discussion amongst the study team.

### How Doulas support (HDS) birthing people of color

Twelve studies focused on how doulas support birthing people of color [21, 22, 24, 27–35] (Table 2: How Doulas Support). All studies were qualitative. Four studies investigated birthing people of color’s perspectives of doula services [22, 24, 27, 33]. Five of the twelve studies focused on doulas with experience working with underserved populations [28–30, 32, 35] and two studies focused on the perspectives of doulas and birthing people of color [31, 34]. Only two of the twelve studies addressed adolescent birthing people of color [31, 33]. Among these studies, we identified four themes: (1) agency and empowerment, (2) relationship building, (3) education, and (4) advocacy. It should be noted that there was often overlap among articles, such that some articles addressed multiple themes.

### Agency and empowerment

Articles embodying this theme (5/12) often articulated the ways in which doulas helped their clients own their autonomy in healthcare decision-making, particularly as it related to their desired birthing experience [24, 27, 29, 30, 32]. In these studies, participants were asked about their birthing experiences and/or perceptions of doulas and doula care. In one study, for example, birthing people of color shared that doulas helped them establish agency by facilitating an environment that helped them to feel more secure and confident in their abilities to voice their desires and concerns and garner respect from their healthcare team [24]. Specifically, doulas were described as sources of social support, as they shared information with their clients concerning the birthing process, certain birth-related practices, and other healthcare-related information that enabled their clients to feel more knowledgeable and prepared during interactions with their healthcare team thereby reinforcing birthing people’s rights to shape the type of birthing experience that they preferred [24].

### Relationship building

Doulas built relationships with clients by establishing trust and communicating effectively to maximize comfort during labor and birth (6/12) [22, 27–29, 31, 32]. Participants were asked about labor and birth experiences of themselves or that of their clients, access to quality prenatal care, and degree of social network support. One study that interviewed doulas of color, most of whom identified as Black/African Americans, found that doulas often perceived themselves as liaisons between clients and their providers, ensuring that their clients’ voices were heard and requests, respected [29]. Doulas serving low-income birthing people of color, as reported in another study, believed that sharing similar racial and/or cultural backgrounds with their clients enabled them to develop stronger relationships, making it easier for them to support their clients during interactions with medical providers [28].

### Education

Doulas’ served as teachers and educators, informing their clients about birthing options, best practices regarding infant caregiving, the labor and birth process, strategies for coping with the prenatal, perinatal, and postpartum periods, and any health-related details specific to the client that may impact their birth experience (6/12) [21, 22, 24, 30, 32, 33]. The focus of questions for birthing people and doulas were different; for birthing people, questions tended to focus on the quality of their birthing experiences while for doulas, questions tended to focus on perceived roles in serving their communities, as well as their training and work experiences in healthcare settings. For example, one study conducted focus groups with pregnant people, most of whom were people of color (63%), who shared their perception of doulas as health educators, or support professionals who provided them with important information and guidance to assist them with managing their pregnancies and proceeding to the perinatal and postpartum periods [21]. In a study focused on the perceptions of doulas, participants shared that they, as doulas, are responsible for addressing other health and social needs of their clients, particularly when such needs could impact birthing people’s abilities to care for themselves and their children [32]. For example, doulas may educate their clients about the availability of resources to support them, equipping them with information that could assist them with addressing their needs [32].

### Advocacy

Doulas assisted their clients by voicing their clients’ rights to quality care in healthcare settings and helping to reduce barriers to quality care through decisive action (6/12) [21, 22, 30, 32, 34, 35]. Generally, birthing people of color were asked about their perceptions of the medical care they were currently receiving or had received during pregnancy. For doulas, questions tended to focus on their roles and responsibilities to their clients during pregnancy and labor and birth, as well as their perceptions of their clients’ needs for various services and resources. For example, in one study, doulas working with Karen refugees who were either pregnant or had just given birth shared their perceived responsibility to advocate for their clients, leveraging their knowledge of their clients’ perinatal preferences and concerns to advocate for quality medical care [34]. In another study, doulas spoke about their obligation to reduce racial inequities by mitigating instances of discrimination observed during patient-provider interactions [32]. Together, these studies highlight doulas’ roles as patient advocates, which is reflected in the curricula for some doula training programs that emphasize training on racial and reproductive justice, health inequities, and the impacts of structural racism on pregnancy and birth outcomes [35].

### Doula Support outcomes (DSO)

Nine quantitative studies focused on pregnancy and/or birthing outcomes associated with utilizing doula support [36–45] (Table 3: Doula Support Outcomes). These studies utilized diverse study designs, including a randomized controlled trial (*n* = 1) [37], retrospective data analyses (*n* = 5) [39–41, 43, 44], pre- and post-intervention comparison analysis (*n* = 2) [38, 42], and a non-randomized, unmasked intervention (quasi-experimental) (*n* = 1) [36].

Seven studies considered measurable birth outcomes related to maternal and infant health including, but not limited to, preterm birth, cesarean delivery, breastfeeding initiation, and epidural and pain medication usage associated with engagement in doula support services [36–39, 41–44]. One study examined infant learning and early parenting behaviors of the birthing people or caregivers [37, 45] while another study examined doulas’ perceptions of the degree to which healthcare practitioners provided respectful care to their patients in the presence of doulas [40]. Only four studies reported racial/ethnic differences in engagement of doula support services [39–41, 43].

#### Preterm birth

Six of nine articles in the DSO category compared the rates of preterm birth by receipt of doula support services [37, 39, 41–44]. All six articles focused on doula outcomes among birthing people from low-income backgrounds who were on Medicaid. Of these, four concluded that there were no significant differences in preterm birth rates between those who received support from doulas compared to their counterparts [37, 41, 43, 44]. Data reported in two articles, however, suggested that utilization of doula support services was associated with lower rates of preterm births [39, 42].

#### Cesarean births

Six of nine articles compared the rates of cesarean births by receipt of doula support services [37, 39, 41–44]. Of these, four studies concluded that there were no significant differences in cesarean birth rates between those who received support from doulas compared to their counterparts [37, 41, 42, 44]. While the remaining two studies found that engagement in doula support services was associated with lowered rates of cesarean births, there were some differences between racial/ethnic groups among people of color [39, 43]. For example, one study found that Black/African American women on Medicaid who received doula support services were more likely than their white counterparts to experience cesarean birth at full gestation whereas Asian and Hispanic women had comparatively lower rates of cesarean births [39]. Another study examined the impact of including doulas in the healthcare team on birthing outcomes among birthing people with limited access to resources and found that receipt of doula support services was not associated with a change in the rates of cesarean births among Black/African American birthing people [43].

#### Breastfeeding initiation

Six of nine articles examined the associations between engaging in doula support services and breastfeeding initiation, knowledge, or intentions among birthing people of color [36–38, 41, 43, 44]. Of these, five were interventions studies that compared behavioral outcomes for birthing people who engaged in doula support services [36–38, 41, 43]. All five studies focused on outcomes among birthing people from low-income backgrounds, with some studies including refugees and non-English speakers. These studies found that breastfeeding initiation and intention was higher among birthing people of color who engaged doulas, with one study indicating a ten-fold increase compared to the control group [43]. However, there were no racial differences in outcomes when comparing Black/African American and white birthing people [43]. One study, however, found that birthing people from vulnerable backgrounds (e.g., refugees, non-English speakers, teens, low income, low education) who received doula support services had lower rates of breastfeeding initiation than their peers from non-vulnerable backgrounds [44].

#### Epidural and other pain medication usage

Three of nine articles examined the association between the utilization of doula support services and the use of an epidural and other pain medications during labor [37, 43, 44]. One was an intervention study comparing the outcomes of two groups of participants, those who received doula support services and those who received standard of care and case management [37]. The other two studies used retrospective data to evaluate the effect of doula support on birthing outcomes, including the use of epidural and pain medication during labor and birth and found that those in the doula care arm had a significantly lower rate of epidural and other pain medication usage during birth [43, 44].

#### Early parenting behaviors

One of nine articles explored associations among the utilization of doula support services and positive parenting practices, finding that birthing people in a doula-home-visit intervention group were more likely than their peers in a control group to begin activities aimed at fostering their infant’s cognitive development, and implementing child safety practices [37].

#### Respectful care

One study examined the relationship between the presence of a doula and respectful care or treatment during labor and birth, particularly for historically marginalized and minoritized birthing people [40]. The results showed a higher odds of receiving respectful care among birthing people with doula support (40% higher) when compared to their peers without doula support [40].

### Considerations for implementing Doula Support (CIDS)

We identified four articles illuminating key considerations when implementing a doula support program [45–48] (see Table 4). First, it was notable that the utilization of doula support services was relatively low amongst birthing people of color. Generally, the reasons for low engagement with these professionals were described as multifaceted and included factors such as low awareness of the doula profession and doulas’ roles in the prenatal, perinatal, and postpartum process. One study, for example, found that only 36.8% of Black/African American women were knowledgeable about the roles of doulas compared to 85.7% of white women [48]. Specifically, sociodemographic factors such as, being Black/African American, being pregnant as a teen, living in extreme poverty, having lower levels of educational attainment, residence in a high-crime neighborhood, not having private insurance, and not being partnered, reduced the odds that a birthing person was aware of doula support services [48].

Second, one study suggested that the uptake of doula support services within communities of color may be improved through strategic partnerships. Specifically, greater engagement in a community-based doula support program was attributed to establishing positive relationships with hospitals, as these efforts helped integrate doulas into the labor and birth unit and increased client referrals to the doula program [46]. This study also highlighted the significance of racial concordance between doulas and their clients, such that this concordance was linked to greater interest and enrollment in the program [46].

Third, birthing support professionals, including doulas and labor and birth nurses, reported exposure to six types of behaviors exhibited by medical providers that were deemed “disrespectful,” including: verbal threats, microaggressions, sexual harassment, racism and discrimination, poor treatment of clients, and invalidation of their client’s wishes [47]. Witnessing disrespectful care was associated with leaving the doula or nursing profession [47].

Lastly, one study examined the engagement levels of young, Black/African American birthing people enrolled in pregnancy and perinatal doula home visiting program. The engagement level was measured by mothers’ initiative in asking questions, emotional connection with the doula, mastery of materials discussed, and the depth and breadth of conversation with doulas [45]. Study results indicated that longer engagement with doulas had a positive impact on engagement levels [45].

## Discussion

This scoping review aimed to characterize the current literature on doula utilization among birthing people of color, identify gaps in the field, and offer recommendations for future directions. Overall, we found that there were three overarching categories wherein which most articles seeking to characterize or assess doula utilization services within our designated time frame could be classified: How doulas support (HDS), doula support outcomes (DSO), and considerations for implementing doula support services (CIDS).

Within the HDS category, we identified four themes: agency and empowerment, relationship-building, education, and advocacy. Studies within the agency and empowerment theme, for example, highlighted the benefits of including doulas on one’s birthing team, as patients often viewed them as sources of social support and pregnancy and birthing education [24, 30, 32]. From their clients’ perspectives, doulas were seen as professionals who provided their clients with valuable information about the birthing process and helped birthing people feel more confident and comfortable expressing their needs and desires with their healthcare teams (e.g., [24]). However, knowledge of the roles and benefits of doulas varied for clients and clinicians [49–51], with most studies suggesting little knowledge of doulas among many birthing people of color. Lack of knowledge of doulas among potential clients and clinicians has a significant impact on utilization of their services [48, 51]. More efforts are needed to promote awareness of doula support services to determine how best to integrate them within the healthcare team, ensuring that the needs of birthing people of color are met. Moreover, as doulas may have more personal knowledge of birthing patients than their medical providers who often spend a limited amount of time with their patients, they may offer unique insights on the individual impact of socio-structural determinants on client behaviors and outcomes thereby assisting both patients and medical professionals with facilitating a healthy pregnancy, birth, and postnatal period. Thus, increasing knowledge of the role of doulas and how they could support the birthing plan through awareness campaigns, physician referrals and clinical training may increase the inclusion of doulas within healthcare teams and subsequently mitigate tension hindering the patient-provider-doula relationships [52]. With research consistently demonstrating that the concerns of Black/African American birthing people are frequently ignored or dismissed [14, 21, 32], doulas may serve an important role in supporting their clients’ confidence in advocating for themselves and their children; however, research in this area is underdeveloped, making it difficult to determine doulas’ true value to the healthcare team. Regarding advocacy, doulas were identified as key resources for birthing people during the postpartum period [21, 32, 37], often assisting their clients with activities that are often viewed as beyond the scope of their professional responsibilities [28]. While such activities could encourage healthy postpartum recovery practices for birthing people, they may also increase the risk that doulas may experience burnout [28]. As such, doulas working with birthing people with significant social needs may require additional support and resources.

Within the DSO category, articles highlighted the ways in which doulas impact birthing outcomes among BIPOC. Some of the articles in this review found that doula support was associated with better birthing outcomes and childrearing practices [39–41, 43]. These findings suggest that doulas may play important roles in the health and well-being of birthing people of color and their infants. Yet, doulas remain underutilized among these communities. Several studies in our review suggested that birthing people of color were less likely than their white peers to be aware of doula support services and the role of doulas in the birthing process [48]. Given the potential benefits of engaging birthing people of color in doula support services, more efforts are needed to increase awareness of the roles of these professionals, whose efforts may reduce maternal child mortality rates in this population [53].

Findings regarding the impact of doula support on birthing outcomes were mixed, particularly concerning preterm births and cesarean births. Several potential mediating factors were identified, including limitations of the study design and/or sample, lack of racial/ethnic diversity among doulas which could impact trust and engagement, and limitations of the length of doula-client interactions [43]. It is notable that only one of the articles in the review investigated the impact of doula care on mental health outcomes, particularly in relation to postpartum depression. Research suggests that birthing people of color may be more susceptible to postpartum depression [54–56]. Postpartum depression has been linked to poor outcomes related to parental and infant development including parent/infant bonding, increased postpartum smoking and suicidality, infant developmental delays, and infant sleeping and eating problems [57]. Studies have shown that birthing people of color who experience postpartum depression are less likely to seek treatment due to various factors, including mental health-related stigma, distrust of health care providers, and lack of access to culturally competent mental health resources [54, 58]. More research is needed to understand whether doula support moderates postpartum depression among birthing people of color and under what conditions.

Within the CIDS category, findings from our review highlight additional opportunities for future research. For example, some studies suggested that racial/ethnic concordance among birthing people and doulas may be beneficial for certain outcomes [28]. Still, concordant race/ethnicity, alone, is likely insufficient for establishing trusting relationships [28]. Moreover, racial/ethnic matching may not be possible or appropriate in all situations. As such, it is important to ensure that all doulas working with birthing people of color receive advanced training, mentoring, or supervision to support the development cultural competence and humility, and skills, to support better service to and more positive outcomes for their clients. Moreover, additional protective measures may be needed to support doulas own mental health, as well as their abilities to advocate for their clients, given the non-traditional roles that many of them take on in service to their clients. While there are many potential benefits of engaging in doulas support services among birthing people of color, the cost of services may be a barrier to engagement. In the US, private birth doulas are usually hired by birthing people with higher incomes or insurance coverage [59]. While some doulas offer special discounts for their services and others are employed by community-based programs offering free services through special programs [59], insurance coverage for such services is limited [60]. As such, the cost of doula services may reduce access to this potentially useful service among birthing people of color thereby exacerbating racial/ethnic disparities in doula access and engagement, and birthing outcomes.

Findings from this review also highlight questions about doula certification and training requirements. In the US, doulas are not required to receive licensure or certification, and there are no national standards or regulations for doula training [61]. National standards for doula training and certification could act to protect their clients from potential malpractice and strengthen calls to integrate doulas into healthcare teams.

Our review is not without limitations. First, this review utilized only four research databases within a limited search period. As such, it is possible that some relevant studies were omitted from our sample. Still, the results of this scoping review contribute to our understanding of the state of science on this topic and offer suggestions regarding areas of potential growth. Second, because this is a scoping review rather than a meta-analysis focused on birthing people of color, these results may not be generalizable to all populations. Specifically, we focused on community dwelling birthing people of color in the US. As such, we did not include studies of birthing people in other settings (e.g., carceral facilities) of those from other countries, which could reduce the generalizability of results. Furthermore, we did not conduct assessments of study quality or bias, given that our aim in undertaking a scoping review, as opposed to a systematic one, was to highlight gaps in the current literature to inform future research. Additionally, our study focused on research published between 2016 and 2022. Consequently, there is a possibility that pivotal studies were omitted from our review, thus limiting the potential for generalization. Nonetheless, the primary objective of this scoping review was to highlight both the current state of knowledge and the gaps in the field to guide future research directions.

## Conclusion

This review sought to characterize the state of the literature on doula utilization among birthing people of color and identify gaps in the field. Our findings suggest that doulas may serve as importance sources of support for birthing people of color and wider implementation of their services has the potential to reduce disparities associated with the birthing process. However, research in this area remains underdeveloped. Greater attention is needed to advance knowledge regarding best practices for integrating doulas in healthcare teams, and the potential impact of the variability in doulas’ training, preparation, and skills on client/patient outcomes. Moreover, research examining the impact of doula engagement and maternal morbidity and mortality remains understudied, and was rarely addressed in studies included in this review. As such, future research should explore that impact of doula support on maternal postpartum outcomes.

## Data Availability

No datasets were generated or analysed during the current study.

## Abbreviations

BIPOC: Black, Indigenous, People of Color
US: United States
CIDS: Considerations for Implementing Doula Support
HDS: How Doulas Support
DSO: Doula Support Outcomes
PRISMA: Preferred Reporting Items for Systematic Reviews and Meta-Analyses
CINAHL: Cumulative Index to Nursing and Allied Health Literature
MeSH: Medical Subject Headings

## References

[1] Chambers BD, Erausquin JT, Tanner AE, Nichols TR, Brown-Jeffy S (2018). Testing the association between traditional and novel indicators of county-level structural racism and birth outcomes among black and white women. J Racial Ethnic Health Disparities.

[2] Bornstein E, Eliner Y, Chervenak FA, Grünebaum A (2020). Racial disparity in pregnancy risks and complications in the US: temporal changes during 2007–2018. J Clin Med.

[3] Jiang L, Tang K, Magee LA, von Dadelszen P, Ekeroma A, Li X (2022). A global view of hypertensive disorders and diabetes mellitus during pregnancy. Nat Reviews Endocrinol.

[4] Shahul S, Tung A, Minhaj M, Nizamuddin J, Wenger J, Mahmood E (2015). Racial disparities in comorbidities, complications, and maternal and fetal outcomes in women with preeclampsia/eclampsia. Hypertens Pregnancy.

[5] Petersen EE, Davis NL, Goodman D, Cox S, Syverson C, Seed K (2019). Racial/ethnic disparities in pregnancy-related deaths—United States, 2007–2016. Morb Mortal Wkly Rep.

[6] Center for Disease Control and Prevention. Racial/Ethnic Disparities in Pregnancy-Related Deaths — United States, 2007–2016 2022 [ https://www.cdc.gov/reproductivehealth/maternal-mortality/disparities-pregnancy-related-deaths/infographic.html.

[7] Manuck TA, editor. Editor racial and ethnic differences in preterm birth: a complex, multifactorial problem. Seminars in perinatology. Elsevier; 2017. 10.1053/j.semperi.2017.08.010 PMC6381592 28941962

[8] Johnson JD, Green CA, Vladutiu CJ, Manuck TA (2020). Racial disparities in prematurity persist among women of high socioeconomic status. Am J Obstet Gynecol MFM.

[9] Hill L, Artiga S, Ranji U. Racial Disparities in maternal and infant health: current status and efforts to address them: Kaiser family foundation; 2022 [ https://www.kff.org/racial-equity-and-health-policy/issue-brief/racial-disparities-in-maternal-and-infant-health-current-status-and-efforts-to-address-them/#:~:text=Infants%20born%20to%20Black%2C%20AIAN,disparities%20widened%20for%.

[10] Njoku A, Evans M, Nimo-Sefah L, Bailey J (2023). Listen to the whispers before they become screams: addressing black maternal morbidity and mortality in the United States.

[11] van Daalen KR, Kaiser J, Kebede S, Cipriano G, Maimouni H, Olumese E (2022). Racial discrimination and adverse pregnancy outcomes: a systematic review and meta-analysis. BMJ Global Health.

[12] Lu MC, Kotelchuck M, Hogan V, Jones L, Wright K, Halfon N (2010). Closing the Black-White gap in birth outcomes: a life-course approach. Ethn Dis.

[13] Salm Ward TC, Mazul M, Ngui EM, Bridgewater FD, Harley AE (2013). You learn to go last: perceptions of prenatal care experiences among African-American women with limited incomes. Matern Child Health J.

[14] Vedam S, Stoll K, Taiwo TK, Rubashkin N, Cheyney M, Strauss N (2019). The giving Voice to Mothers study: inequity and mistreatment during pregnancy and childbirth in the United States. Reproductive Health.

[15] Taylor JK (2020). Structural racism and maternal health among black women. J Law Med Ethics.

[16] Wallace M, Crear-Perry J, Richardson L, Tarver M, Theall K (2017). Separate and unequal: structural racism and infant mortality in the US. Health Place.

[17] Crear-Perry J, Correa-de-Araujo R, Lewis Johnson T, McLemore MR, Neilson E, Wallace M (2021). Social and structural determinants of health inequities in maternal health. J Women’s Health.

[18] Wang E, Glazer KB, Howell EA, Janevic TM (2020). Social determinants of pregnancy-related mortality and morbidity in the United States: a systematic review. Obstet Gynecol.

[19] Valerio VC, Downey J, Sgaier SK, Callaghan WM, Hammer B, Smittenaar P. Black-white disparities in maternal vulnerability and adverse pregnancy outcomes: an ecological population study in the United States, 2014–2018. Lancet Regional Health–Americas. 2023;20. 10.1016/j.lana.2023.100456 PMC10122115 37095772

[20] Collins CC, Brown PL, Rice H, Bronson C, Cherney E, Farmer C, DeRigne L (2021). Experiences of black women during pregnancy: the meaning of perinatal support. Am J Orthopsychiatry.

[21] Attanasio LB, DaCosta M, Kleppel R, Govantes T, Sankey HZ, Goff SL (2021). Community perspectives on the creation of a hospital-based doula program. Health Equity.

[22] Hansen MED, James BA, Sakinah I, Speights JSB, Rust G (2021). Traversing traditions: prenatal care and birthing practice preferences among black women in North Florida. Ethn Dis.

[23] Phillips-Bell G. Collaboration between maternal and child health and chronic disease epidemiologists to identify strategies to reduce hypertension-related severe maternal morbidity. 2019. 10.5888/pcd16.190045 PMC6936670 31831105

[24] Kozhimannil KB, Vogelsang CA, Hardeman RR, Prasad S (2016). Disrupting the pathways of social determinants of health: doula support during pregnancy and childbirth. J Am Board Family Med.

[25] Tricco AC, Lillie E, Zarin W, O’Brien KK, Colquhoun H, Levac D (2018). PRISMA extension for scoping reviews (PRISMA-ScR): checklist and explanation. Ann Intern Med.

[26] Levac D, Colquhoun H, O’Brien KK (2010). Scoping studies: advancing the methodology. Implement Sci.

[27] Collins C, Bai R, Brown P, Bronson CL, Farmer C (2023). Black women’s experiences with professional accompaniment at prenatal appointments. Ethn Health.

[28] Wint K, Elias TI, Mendez G, Mendez DD, Gary-Webb TL (2019). Experiences of community doulas working with low-income, African American mothers. Health Equity.

[29] Kathawa CA, Arora KS, Zielinski R, Low LK (2022). Perspectives of doulas of color on their role in alleviating racial disparities in birth outcomes: a qualitative study. J Midwifery Women’s Health.

[30] Guerra-Reyes L, Hamilton LJ (2017). Racial disparities in birth care: exploring the perceived role of African-American women providing midwifery care and birth support in the United States. Women Birth.

[31] Coley SL, Nichols TR (2016). Understanding factors that influence adolescent mothers’ doula use: a qualitative study. J Perinat Educ.

[32] Kett PM, van Eijk MS, Guenther GA, Skillman SM (2022). This work that we’re doing is bigger than ourselves: a qualitative study with community-based birth doulas in the United States. Perspect Sex Reprod Health.

[33] Hans SL, Cox SM, Medina NY (2022). African American adolescent mothers’ Childbirth Support from fathers, grandmothers, nurses, doctors, and Doulas. J Perinat Educ.

[34] LaMancuso K, Goldman RE, Nothnagle M (2016). Can I ask that? Perspectives on perinatal care after resettlement among Karen refugee women, medical providers, and community-based doulas. J Immigr Minor Health.

[35] Van Eijk MS, Guenther GA, Kett PM, Jopson AD, Frogner BK, Skillman SM (2022). Addressing systemic racism in birth doula services to reduce health inequities in the United States. Health Equity.

[36] Furman L, Matthews L, Davis V, Killpack S, O’Riordan MA (2016). Breast for success: a community–academic collaboration to increase breastfeeding among high-risk mothers in Cleveland. Progress Community Health Partnerships: Res Educ Action.

[37] Hans SL, Edwards RC, Zhang Y (2018). Randomized controlled trial of doula-home-visiting services: impact on maternal and infant health. Matern Child Health J.

[38] Louis-Jacques AF, Vereen S, Hernandez I, Običan SG, Deubel TF, Miller EM (2021). Impact of Doula-Led lactation education on breastfeeding outcomes in low-income, minoritized mothers. J Perinat Educ.

[39] Kozhimannil KB, Hardeman RR, Alarid-Escudero F, Vogelsang CA, Blauer‐Peterson C, Howell EA (2016). Modeling the cost‐effectiveness of doula care associated with reductions in preterm birth and cesarean delivery. Birth.

[40] Mallick LM, Thoma ME, Shenassa ED (2022). The role of doulas in respectful care for communities of color and Medicaid recipients. Birth.

[41] Mosley EA, Pratt M, Besera G, Clarke LS, Miller H, Noland T et al. Evaluating birth outcomes from a community-based pregnancy support program for refugee women in Georgia. Front Global Women’s Health. 2021:37. 10.3389/fgwh.2021.655409 PMC8593936 34816209

[42] Thomas M-P, Ammann G, Brazier E, Noyes P, Maybank A (2017). Doula services within a healthy start program: increasing access for an underserved population. Matern Child Health J.

[43] Thurston LAF, Abrams D, Dreher A, Ostrowski SR, Wright JC (2019). Improving birth and breastfeeding outcomes among low resource women in Alabama by including doulas in the interprofessional birth care team. J Interprofessional Educ Pract.

[44] Van Zandt SE, Kim S, Erickson A (2016). Nursing student birth doulas’ influence on the childbearing outcomes of vulnerable populations. J Commun Health Nurs.

[45] Wen X, Korfmacher J, Hans SL (2016). Change over time in young mothers’ engagement with a community-based doula home visiting program. Child Youth Serv Rev.

[46] Marshall C, Arteaga S, Arcara J, Cuentos A, Armstead M, Jackson A, Manchikanti Gómez A (2022). Barriers and facilitators to the implementation of a community doula program for Black and Pacific Islander pregnant people in San Francisco: findings from a partnered process evaluation. Matern Child Health J.

[47] Morton CH, Henley MM, Seacrist M, Roth LM (2018). Bearing witness: United States and Canadian maternity support workers’ observations of disrespectful care in childbirth. Birth.

[48] Sperlich M, Gabriel C (2019). St. Vil NM. Preference, knowledge and utilization of midwives, childbirth education classes and doulas among US black and white women: implications for pregnancy and childbirth outcomes. Soc Work Health Care.

[49] Adams C, Curtin-Bowen M (2021). Countervailing powers in the labor room: the doula–doctor relationship in the United States. Soc Sci Med.

[50] Neel K, Goldman R, Marte D, Bello G, Nothnagle MB (2019). Hospital-based maternity care practitioners’ perceptions of doulas. Birth.

[51] Turner D, Lindsey A, Shah P, Sayyad A, Mack A, Rice WS, Mosley EA (2022). Doulas shouldn’t be considered visitors, we should be considered a part of [the] team: doula care in Georgia, USA during the COVID-19 pandemic. Sex Reproductive Health Matters.

[52] Horstman HK, Anderson J, Kuehl RA (2017). Communicatively making sense of doulas within the US master birth narrative: Doulas as liminal characters. Health Commun.

[53] Bohren MA, Hofmeyr GJ, Sakala C, Fukuzawa RK, Cuthbert A. Continuous support for women during childbirth. Cochrane Database Syst Rev. 2017(7). 10.1002/14651858.CD003766.pub6 PMC6483123 28681500

[54] Cannon C, Nasrallah H (2019). A focus on postpartum depression among African American women: a literature review. Annals Clin Psychiatry: Official J Am Acad Clin Psychiatrists.

[55] Gopalan P, Spada ML, Shenai N, Brockman I, Keil M, Livingston S (2022). Postpartum Depression—identifying risk and Access to intervention. Curr Psychiatry Rep.

[56] Guintivano J, Manuck T, Meltzer-Brody S (2018). Predictors of postpartum depression: a comprehensive review of the last decade of evidence. Clin Obstet Gynecol.

[57] Slomian J, Honvo G, Emonts P, Reginster J-Y, Bruyère O (2019). Consequences of maternal postpartum depression: a systematic review of maternal and infant outcomes. Women’s Health.

[58] Bossick AS, Bossick NR, Callegari LS, Carey CM, Johnson H, Katon JG (2022). Experiences of racism and postpartum depression symptoms, care-seeking, and diagnosis. Arch Women Ment Health.

[59] Gomez AM, Arteaga S, Arcara J, Cuentos A, Armstead M, Mehra R (2021). My 9 to 5 job is birth work: a case study of two compensation approaches for community doula care. Int J Environ Res Public Health.

[60] Kozhimannil KB, Hardeman RR (2016). Coverage for doula services: how state Medicaid programs can address concerns about maternity care costs and quality. Birth (Berkeley Calif).

[61] Henley MM (2015). Alternative and authoritative knowledge: the role of certification for defining expertise among doulas. Social Currents.

